# Dolutegravir use over 48 weeks is not associated with worsening insulin resistance and pancreatic beta cell function in a cohort of HIV-infected Ugandan adults

**DOI:** 10.21203/rs.3.rs-3175598/v1

**Published:** 2023-07-20

**Authors:** Frank Mulindwa, Barbara Castelnuovo, Nele Brusselaers, Martin Nabwana, Robert Bollinger, Eva Laker, Ronald Kiguba, Jean-Marc Schwarz

**Affiliations:** Makerere University Infectious Diseases Institute; Makerere University Infectious Diseases Institute; Antwerp University; Makerere University - Johns Hopkins University Research Collaboration; Johns Hopkins University; Makerere University Infectious Diseases Institute; Makerere University; University of California San Francisco

**Keywords:** Dolutegravir, Insulin resistance, pancreatic beta cell function, HOMA, type 2 Diabetes Mellitus

## Abstract

**Background:**

The Uganda Ministry of Health issued restrictive guidelines on the use of dolutegravir (DTG) in persons stratified to have a heightened risk of diabetes mellitus. This followed multiple reports of persons with HIV (PWH) presenting with accelerated hyperglycemia after a few weeks to months of exposure to DTG. Having demonstrated a low incidence of diabetes mellitus and improving blood glucose trajectories in a cohort of ART naïve Ugandan PWH on DTG, we sought to determine whether the observed improvement in blood glucose did not mask background compensated insulin resistance.

**Methods:**

In this analysis, 63 patients underwent serial oral glucose tolerance tests over 48 weeks. Using fasting serum insulin and glucose, we calculated insulin resistance and pancreatic beta cell function by homeostatic modelling (HOMA IR and HOMA%β respectively). Absolute mean changes between baseline and post-baseline blood glucose, pancreatic beta cell function and insulin resistance were computed by subtracting each post-baseline value from the baseline value and compared using student t-test. Multiple linear regression models were used to determine the factors associated with changes in pancreatic beta cell function and insulin resistance.

**Results:**

Of the 63 participants, 37 (58%) were female. Median age was 31 (IQR: 28–37). Despite a trend towards an initial increase in both HOMA IR and HOMA%β at 12 weeks followed by a decline through 36 weeks to 48 weeks, the HOMA IR and HOMA%β at 48 weeks were not significantly different from baseline i.e. (difference in mean HOMA IR from baseline: 0.14, 95%CI: −0.46, 0.733, p = 0.648) and (difference in mean HOMA %β from baseline: 6.7, 95%CI: −13.4, 26.8, p = 0.506) respectively.

## Introduction

Following reports of wide spread primary resistance to non-nucleoside reverse transcriptase inhibitors (NNRTIs), the World Health Organization (WHO) recommended dolutegravir, an integrase strand transfer inhibitor (INSTI) as an anchor drug for first line and later second line treatment of HIV[1]–[4]. In multiple studies, dolutegravir had demonstrated very high efficacy, a high genetic barrier to resistance as well as a very good side effect profile[5], [6]. In the years that followed the WHO recommendations, DTG was widely adopted in majority HIV treatment programs in sub-Saharan Africa as first line therapy[7], [8].

Uganda adopted DTG anchored anti-retroviral therapy (ART) in 2018[9], [10]. Selected HIV treatment centers such as the Makerere University Infectious Diseases Institute acted as pilot sites. ART naïve PWH were initiated on DTG with concurrent switching of ART-experienced PWH. In the first 12 months of use, the Makerere University Infectious Diseases Institute reported sixteen cases of diabetic ketoacidosis happening weeks to a few months after switching to DTG, the majority of whom were ART experienced prior to the switch to DTG[11]. Multiple anecdotal reports followed, prompting the Uganda Ministry of Health to issue restrictive guidelines on DTG use including: avoiding the use of DTG in PWH known to have diabetes mellitus (DM) and three-monthly monitoring of blood glucose for PWH with pre-diabetes mellitus at baseline[12]. Much as the reported events were mainly in ART experienced patients, the guidelines applied to ART naïve patients as well.

We explored the relationship of INSTI use with incident diabetes in PWH at population level in an earlier meta-analysis[13], [14]. In that analysis, we demonstrated that the risk of incident DM is actually reduced with INSTI use compared to protease inhibitors and NNRTIs apart from African populations which were largely under-represented. Following that publication, we sought to determine the incidence of DM in an African setting (Ugandan ART naïve PWH on DTG for 48 weeks) using serial oral glucose tolerance tests[15]. We determined that the incidence was very low, less than in most safety data reported from DTG landmark trials and large population cohorts[15]. Fasting and 2-hour blood glucose trajectories also demonstrated a trend towards improvement over 48 weeks. Most participants with incident pre-diabetes mellitus had transient hyperglycemia that resolved on prospective assessments.

Before onset of pre-diabetes or overt DM, mechanisms such as insulin hyper-secretion and reduced renal insulin clearance may effectively compensate for insulin resistance[16], [17]. This means, changes in blood glucose may lag behind changes in insulin resistance as well as pancreatic beta cell failure. It is possible the reassuring blood glucose trajectories we demonstrated may mask early stages of worsening insulin resistance and pancreatic beta cell function. Therefore, we assessed pancreatic beta cell function and insulin resistance patterns in the same cohort of Ugandan PWH on DTG for 48 weeks.

## METHODS

### Study design and setting

The GLUMED study was a prospective cohort study at the Kisenyi Health Center IV, HIV clinic in Uganda’s capital city, Kampala. The clinic is supported by the IDI with funding from the Center for Disease Control (CDC) and the U.S. President’s Emergency Plan for AIDS Relief (PEPFAR). Ugandan PWH were recruited between 1st - January- 2021 and 20th -October − 2021 and followed up to the end of September- 2022.

#### Study participants and study processes.

ART naïve PWH aged ≥ 18 years enrolling for ART care were screened for study inclusion. Pregnant women and patients unable to undergo a 2 hour - 75g oral glucose tolerance test (2h-OGTT) were excluded. Criteria for exclusion during follow up included: new pregnancy and poor adherence to ART (adherence < 85% determined by pill count and self-reporting[12]).

After providing informed consent, patients were scheduled for review in one to two days (whichever was convenient to them) after an overnight fast of 8–12 hours. Baseline demographic, clinical and social data were collected. A 2-hour oral glucose tolerance test (2h-OGTT) was performed[18]. Patients found not to have diabetes mellitus on the 2h-OGTT (fasting blood glucose (FBG) < 126mg/dl and 2-hour blood glucose (2hBG) < 200mg/dl) were enrolled for 48-week follow up on tenofovir/ lamivudine/ dolutegravir (TDF/3TC/DTG) in line with the Uganda National HIV treatment guidelines[12]. Enrolled patients received the same adherence and positive living counselling package as the other patients in the Kisenyi HIV clinic before ART initiation.

Repeat 2h- OGTT was performed at 12 and 36 weeks while FBG was measured at 24 and 48 weeks. Viral load monitoring was performed at 24 weeks. ART adherence was evaluated on every clinical visit using self-reports and pill counts as recommended by the Uganda MoH guidelines[19]. Further details of the study processes are reported in our earlier publication[15].

The first 63 participants that completed 48 weeks of follow up were evaluated for this analysis. An online calculator was used to calculate pancreatic beta cell functioning and insulin resistance by homeostatic modeling (HOMA %β and HOMA IR respectively) as a factor of fasting blood glucose and fasting serum insulin[20].

### Outcomes

The primary outcome for this analysis was mean change in pancreatic beta cell function and insulin resistance calculated using the Homeostatic Model (HOMA)[21], [22] from baseline to 48 weeks.

### Statistical analysis

Data were entered in Microsoft Excel 2016, cleaned, and transformed before it was exported for statistical analyses in Stata Release 17.0 software. Generally, data were presented using tables and figures. Continuous variables were presented using medians and their corresponding inter-quartile ranges (IQR). Categorical variables were presented using percentages.

Absolute mean change between baseline and post-baseline blood glucose, pancreatic beta cell function and insulin resistance was computed by subtracting each post-baseline value from the baseline value. Mean change between baseline and post-baseline blood glucose, pancreatic beta cell function and insulin resistance was compared using student t-test. Mean differences (95% Confidence Interval, CI) were reported. Multiple linear regression models were used to determine the factors associated with changes in pancreatic beta cell function and insulin resistance from baseline to 48 weeks. Statistical significance was tested at a p-value of less than 0.05 and all p-values were two-sided.

## Results

### Baseline demographic and clinical characteristics of the study participants

Of the 63 patients analyzed, 37 (58%) were female. The median age of the participants was 31 years (Interquartile range (IQR):28,37) with a median CD4 cell count of 284 cells/mm^3^ (IQR 158,518). Fifty-two (83%) of the patients had a normal blood pressure, 8 (12%) had pre-hypertension and 3 (4%) had hypertension. Sixty-one (97%) of the participants were in WHO HIV clinical stage 1, one patient in clinical stage 2 and one in clinical stage 3. Fifty-two (83%) of the patients had no tuberculosis (TB) symptoms, 10 (16%) had symptoms suggestive of TB but were found not to have active TB disease on evaluation and one patient had an established diagnosis of TB at baseline and treatment was initiated. On weight evaluation, 41 (65%) had a normal body mass index (BMI), 7 (11%) were underweight, 14 (22%) were overweight and one, obese. Fifty-five (87%) met the WHO physical activity requirements[23]. All patients were anti- glutamic acid decarboxylase (anti-GAD) antibody negative while 2 (3%) were positive for anti-Islet cell antigen-2 (anti-IA2) antibodies. All evaluated patients had virologic suppression at 24 weeks. The median serum creatine, fasting LDL, fasting HDL and fasting total cholesterol at baseline were: 0.86 (IQR; 0.77, 0.98), 75.2 (IQR; 53.8, 90.3), 29.6 (IQR: 24.9, 35.8) and 130.9 (IQR:111.2, 158.7) respectively. (Table 1)

#### Changes in mean fasting blood glucose and 2-hour blood glucose from baseline.

There was an initial significant drop in fasting blood glucose at week 12 (difference in mean fasting blood glucose from baseline (FBG): −3.3, 95%CI: −6.0, −0.5), p = 0.020). There after blood glucose leveled off with insignificant changes through 24 and 36 weeks. Fasting blood glucose at 48 weeks was not significantly different from fasting blood glucose at baseline (FBG: 0.4, 95%CI: −2.2, 3.1, p = 0.742).

There was significant reduction in 2hBG at 12 weeks (difference in mean 2hBG from baseline (2hBG): −14.6, 95%CI: −22.1, −7.0, p = 0.0003). thereafter there was an insignificant increase in 2hBG to week 36. 2hBG at 36 weeks was not significantly different from baseline (2Hbg: −5.1, 95%CI: −12.4, 2.1, p = 0.163).

#### Changes in mean pancreatic beta cell function determined by homeostatic modeling (HOMA %β) from baseline.

**Figures 1:**
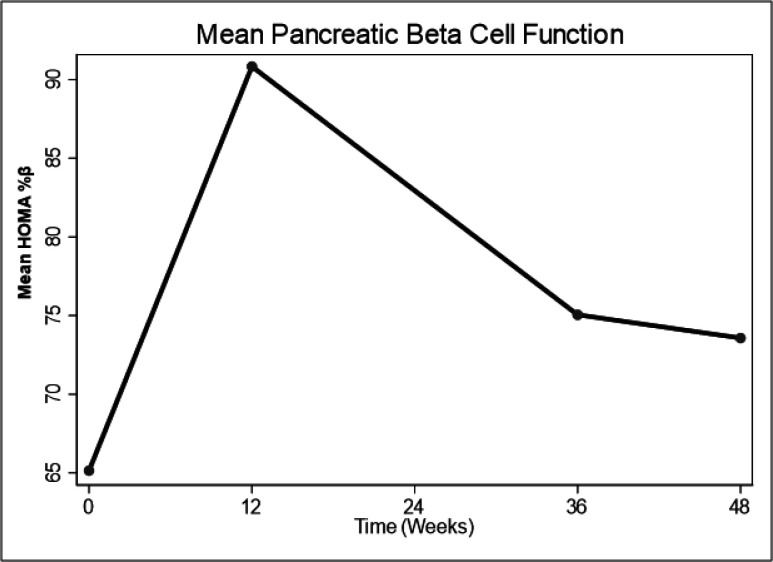
Changes in mean pancreatic beta cell function (HOMA%β) over 48 weeks.

There was a significant increase in HOMA %β at 12 weeks (difference in mean HOMA %β from baseline (HOMA %β): 24.9, 95%CI: 6.3, 43.6, p = 0.01) with subsequent reduction through 36 to 48 weeks but not reaching baseline values. There was no significant difference between HOMA %β at 36 and 48 weeks from baseline i.e. (HOMA %β: 8.8, 95%CI: −13.2, 30.7, p = 0.427) and (HOMA %β: 6.7, 95%CI: −13.4, 26.8, p = 0.506) respectively (Table 2, Fig. 1). Changes in HOMA %β over 48 weeks were independent of factors known to influence glucose metabolism such as: Age, Baseline CD4, Waist circumference, Sex, BMI and physical activity (Table S1- supplementary material).

#### Changes in mean insulin resistance determined by homeostatic modeling (HOMA IR) from baseline.

**Figure 2:**
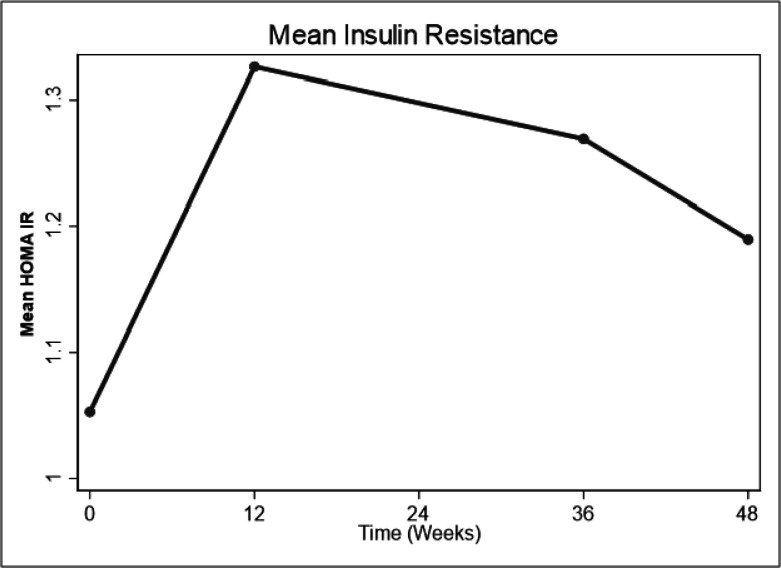
Changes in mean insulin resistance (HOMA IR) over 48 weeks.

There was a trend towards an increase in HOMA IR at 12 weeks with subsequent reduction thereafter through 36 weeks to 48 weeks without reaching baseline values. However, HOMA IR at 12, 36 and 48 weeks was not significantly different from that at baseline i.e. (difference in mean HOMA IR from baseline (HOMA IR):0.27, 95%CI: −0.24, 0.79, p = 0.294), (HOMA IR:0.22, 95%CI: −0.44, 0.87, p = 0.511) and (HOMA IR: 0.14, 95%CI: −0.46, 0.733, p = 0.648) respectively. (Table 2, Fig. 2). Changes in HOMA IR over 48 weeks were as well not influenced by baseline age, baseline CD4, Waist circumference, Sex, BMI and physical activity (Table S1- supplementary material).

## Discussion

We sought to determine changes in insulin resistance (HOMA IR) and pancreatic beta cell function (HOMA%β) over the first 48 weeks of dolutegravir based anti-retroviral therapy in a cohort of Ugandan ART naive PWH. We had earlier demonstrated consistent improvement in glucose tolerance (2-hour blood glucose) of the whole study cohort through 48 weeks. A subsection of the participants developed incident pre-diabetes mellitus, but this was largely transient with reversion to normal blood glucose on prospective clinic visits. [15]. The aim of the study was to determine if despite the reassuring glucose trends, there wasn’t background compensated worsening insulin resistance. We determined that there were no significant changes in both HOMA IR and HOMA%β over the first 48 weeks on dolutegravir.

Pooled results from two studies with participants from Europe and North America determined that the effect of INSTIs on insulin resistance was not significantly different from that of PIs and NNRTIs over 27 months[13]. Our study reaffirms the low risk of insulin resistance in an ART-naïve African population receiving DTG.

In our study, much as insignificant, there was a paradoxical increase in insulin resistance in the first 12 weeks. With immune reconstitution, there is usually better insulin signaling at end organs which improves insulin resistance, a phenomenon that would have been expected on introduction of ART[24], [25]. This initial increase in HOMA IR however could be explained by weight gain and improved appetite on ART introduction. The initial improvement in pancreatic beta cell function at 12 weeks could be explained by immune reconstitution, reduced inflammation on introduction of ART but could as well be compensatory for the initial worsening insulin resistance at 12 weeks[26], [27].

In the reported cases of accelerated hyperglycemia, patients presented less than 48 weeks on integrase inhibitors[11], [28]–[30]. Documenting changes in pancreatic beta cell function and insulin resistance in this initial part of DTG use as demonstrated in this study adds more insight into explaining the pathophysiology behind these events. So far, what is clear is; 1) at population level, INSTIs (to which DTG belongs) are associated with a reduced risk of incident diabetes as compared to other ART drug classes [13], [31]. 2) Most patients presenting with accelerated hyperglycemia are heavily ART experienced before switch to dolutegravir or other integrase inhibitors[11], [28]–[30], [32]. 3) In ART naïve Ugandan patients on dolutegravir, the incidence of diabetes mellitus is very low, comparable to safety data results in landmark DTG trials and largely less than in most cohort studies in Europe and North America[15]. 4) There is a general improvement in blood glucose over 48 weeks in a cohort of ART naïve Ugandan PWH on dolutegravir for 48 weeks[15] and lastly from this study 5) there are insignificant changes in insulin resistance as well as pancreatic beta cell function over 48 weeks in the same cohort of Ugandan ART naïve PWH.

Our study had limitations. We lacked a comparator group hence we could only describe changes in HOMA IR and HOMA%β over the follow up period but couldn’t ascertain to what extent these were due to exposure to DTG. It was a single center study in an urban setting which could limit generalizability of results to the whole Ugandan PWH population. Despite the limitations, we had a clear metabolic outcome and used a programmatic setting allowing participants to have exactly the same ART care as other patients in the study clinic.

## Conclusions

We demonstrated insignificant changes in both insulin resistance and pancreatic beta cell function in Ugandan PWH on dolutegravir for 48 weeks. We add to the body of evidence demonstrating glucose metabolic safety of dolutegravir in ART naïve Ugandan patients. This puts in question restrictive guidelines on the use of dolutegravir in ART naïve Ugandan PWH perceived to have a heightened risk to diabetes.

## Figures and Tables

**Table 1:** Baseline clinical and demographic characteristics of the study participants.

Characteristic	Number (n = 63)	Percentage (%)
**Age**, Median (IQR)	31 (28, 37)	
**Sex**		
Female	37	58.7
Male	26	41.3
**Baseline CD4 cell count**, Median (IQR)	284 (158, 518)	
**Level of education**		
Primary	33	52.4
Secondary	26	41.3
Tertiary	4	6.3
**Religion**		
Christian	49	77.8
Muslim	14	22.2
Residence		
Rural	1	1.6
Urban	62	98.4
**Employment**		
No	9	14.3
Yes	54	85.7
**Marital status**		
Single	33	52.4
Married	30	47.6
**Tuberculosis status at baseline**		
No symptoms	52	82.5
TB suspect	10	15.9
TB disease	1	1.6
**Baseline blood pressure**		
Normal BP	52	82.5
Pre-hypertension	8	12.7
Hypertension	3	4.8
**HIV clinical stage**		
Stage 1	61	96.8
Stage 2	1	1.6
Stage 3	1	1.6
**Body Mass Index (BMI)**		
Underweight (< 18.5)	7	11.1
Normal (18.5–24.9)	41	65.1
Overweight (25.0–29.9)	14	22.2
Obese (≥ 30)	1	1.6
**Waist circumference**		
Normal	44	69.8
Increased risk of cardiometabolic complications	10	15.9
Substantially increased risk of cardiometabolic complications	9	14.3
**Smoking status**		
Smoker	6	9.5
Non-smoker	57	90.5
**Physical activity**		
GPAQ < 600 MET minutes	8	12.7
GPAQ ≥ 600 MET minutes	55	87.3
**Alcohol consumption**		
No consumption	33	52.4
Low risk alcohol consumption	19	30.2
Hazardous alcohol consumption	6	9.5
Risk of alcohol dependence	5	7.9
**24-week viral loads (Proxy baseline VL), n = 62**		
Virologically suppressed	63	100
**Anti-GAD antibody**		
Negative	63	100
**Anti-IA2 antibody status**		
Negative	61	96.8
Positive	2	3.2
**Laboratory investigations, Median (IQR)**		
Creatinine (mg/dl)	0.86 (0.77, 0.98)	
LDL (mg/dl)	75.2 (53.8, 90.3)	
HDL (mg/dl)	29.6 (24.9, 35.8)	
Total cholesterol (mg/dl)	130.9 (111.2, 158.7)	
Triglycerides (mg/dl)	95.2 (71.3, 124.0)	

IQR- interquartile range, BP- blood pressure, GPAQ- Global physical activity questionnaire, MET- metabolic equivalent, anti- GAD- anti- glutamic acid decarboxylase, anti- IA2 - anti- Islet cell antigen-2, LDL- low density lipoproteins, HDL- high density lipoproteins.

**Table 2:** Changes in blood glucose, pancreatic beta cell function and insulin resistance over 48 weeks on dolutegravir in the study participants

Time point	Fasting blood glucose	2-hour OGTT glucose	Pancreatic beta cell function (HOMA %β)	Insulin resistance (HOMA IR)
	Mean (95% CI)	Mean (95% CI)	Mean (95% CI)	Mean (95% CI)
**Baseline**	90.1 (87.6, 92.6)	115.9 (108.8, 122.9)	65.1 (50.6, 79.7)	1.05 (0.54, 1.56)
**Week 12**	86.8 (84.8, 88.8)	101.3 (96.4, 106.2)	90.8 (78.5, 103.2)	1.33 (1.04, 1.61)
Difference	−3.3 (−6.0, −0.5)	−14.6 (−22.1, −7.0)	24.9 (6.3, 43.6)	0.27 (−0.24, 0.79)
p-value	**0.020**	**0.0003**	**0.010**	0.294
**Week 24**	89.2 (87.3, 91.1)			
Difference	−0.8 (−3.3, 1.6)			
p-value	0.497			
**Week 36**	91.2 (89.3, 93.1)	110.4 (105.9, 114.9)	75.1 (61.5, 88.6)	1.27 (0.90, 1.64)
Difference	1.2 (−1.7, 4.1)	−5.1 (−12.4, 2.1)	8.8 (−13.2, 30.7)	0.22 (−0.44, 0.87)
p-value	0.414	0.163	0.427	0.511
**Week 48**	90.5 (88.5, 92.4)		73.6 (61.0, 86.1)	1.19 (0.85, 1.53)
Difference	0.4 (−2.2, 3.1)		6.7 (−13.4, 26.8)	0.14 (−0.46, 0.733)
p-value	0.742		0.506	0.648

OGTT- oral glucose tolerance test, HOMA %β- Homeostatic modeling for pancreatic beta cell function, HOMA IR- Homeostatic modelling for insulin resistance, CI- confidence interval

## Data Availability

The datasets used and/or analyzed during the current study are available from the corresponding author on reasonable request.

## Abbreviations

ART: Anti-retroviral Therapy
NNRTI: Non- Nucleoside Reverse Transcriptase Inhibitors
DTG: Dolutegravir
WHO: World Health Organization
IDI: Infectious Diseases Institute
DKA: Diabetic Ketoacidosis
T2DM: Type 2 Diabetes Mellitus
PLHIV: People Living with HIV
OGTT: Oral Glucose Tolerance Test
CDC: Center for Diseases Control
BMI: Body Mass Index
GPAQ: Global Physical Activity Questionnaire
FBG: Fasting Blood Glucose
2hBG: 2-hour Blood Glucose
MET: Metabolic Equivalent of Task
LDL: Low Density Lipoprotein
HDL: High Density Lipoprotein
